# Observation of strong anisotropic forbidden transitions in (001) InGaAs/GaAs single-quantum well by reflectance-difference spectroscopy and its behavior under uniaxial strain

**DOI:** 10.1186/1556-276X-6-210

**Published:** 2011-03-10

**Authors:** Jin-Ling Yu, Yong-Hai Chen, Chen-Guang Tang, ChongYun Jiang, Xiao-Ling Ye

**Affiliations:** 1 Key Laboratory of Semiconductor Materials Science, Institute of Semiconductors, Chinese Academy of Sciences, P.O. Box 912, Beijing 100083, People's Republic of China

## Abstract

The strong anisotropic forbidden transition has been observed in a series of InGaAs/GaAs single-quantum well with well width ranging between 3 nm and 7 nm at 80 K. Numerical calculations within the envelope function framework have been performed to analyze the origin of the optical anisotropic forbidden transition. It is found that the optical anisotropy of this transition can be mainly attributed to indium segregation effect. The effect of uniaxial strain on in-plane optical anisotropy (IPOA) is also investigated. The IPOA of the forbidden transition changes little with strain, while that of the allowed transition shows a linear dependence on strain.

**PACS **78.66.Fd, 78.20.Bh, 78.20.Fm

## Introduction

It is well known that in-plane optical anisotropy (IPOA) can be introduced in a (001)-grown zinc-blende quantum well (QW) when the symmetry is reduced from *D*_2*d *_to *C*_2υ _[1-6]. There are two kinds of symmetry reduction effect (SRE), one is bulk SRE, and the other is interface SRE [2,4]. The bulk SRE can be introduced by electric field, compositional variation across the QW and uniaxial strain [7-10]. The IPOA induced by uniaxial strain in GaAs/Al*_x_*Ga_1-*x*_As QWs has been reported by Shen [10], Rau [8] and Tang [11]. However, as far as we know, this effect in In*_x_*Ga_1-*x*_As/GaAs QW has never been reported.

The interface SRE, which origins from *C*_2*υ *_symmetry of a (001) zinc-blende interface, can be introduced by special interface chemical bonds, segregation effect and the anisotropic interface structures [2,3,6]. It was found that the interface-induced IPOA was very strong in the QWs sharing no-common-atom, while the IPOA in QWs sharing common atoms such as GaAs/AlGaAs was too weak to be observed by conventional polarized spectroscopy [2,4,10]. Fortunately, the weak IPOA in the AlGaAs/GaAs and InGaAs/GaAs QWs can be well observed by the reflectance-difference spectroscopy (RDS) [2,4,6]. Wang et al. has studied forbidden transitions in In*_x_*Ga_1-*x*_As/GaAs by photoreflectance (PR) and attributed the forbidden transition to the built-in electric field [12]. Chen et al. [1] and Ye et al. [6] observed anisotropic forbidden transition in In*_x_*Ga_1-*x*_As/GaAs by RDS. Chen ascribed the anisotropic forbidden transition to the interplay of interface *C*_2*ν *_symmetry and built-in electric field, while Ye attributed it to both the built-in electric field and segregation effect. In this study, we observed strong anisotropic forbidden transitions in a series of In*_x_*Ga_1-*x*_As/GaAs single-quantum well (SQW) with well width ranging between 3 nm and 7 nm at 80 K. Numerical calculation within the envelope function framework have been performed to analyze the origin of the optical anisotropic forbidden transition. Detailed theory-experiment comparisons show that the anisotropic forbidden transition can be mainly attributed to indium (In) segregation effect. Besides, the effect of uniaxial strain on in-plane optical anisotropy (IPOA) is also investigated. It is found that, the IPOA of the forbidden transition nearly does not change with strain, while that of the allowed transition shows a linear dependence on strain. Finally, an interpretation of the IPOA by perturbation theory is given out.

## Samples and experiments

A series of In_0_._2_Ga_0_._8_As/GaAs SQW with different well widths were grown on (001) semi-insulating GaAs by molecular beam epitaxy. The SQW was sandwiched between two thick GaAs layers. The nominal well widths of the three samples were 3, 5, and 7 nm, respectively. All epilayers were intentionally undoped. The setup of our RDS, described in Ref. [13], is almost the same as Aspnes et al. [14], except the position of the monochromator. The relative reflectance difference between [110] and [110] directions, defined by *Δr/r *= 2(*r*_110 _- *r*_110_)/(*r*_[110] _+ *r*_[110]_), was measured by RDS at 80 K. Here *r*_[110] _(*r*_[110]_) is the reflective index in the [110] ([110]) direction. We also did the reflectance measurements, and thus obtained the *ΔR/R *spectra. Here *R *is the reflectivity of the sample and *ΔR *is the reflectivity difference between samples with and without QW layer.

**Figure 1:**
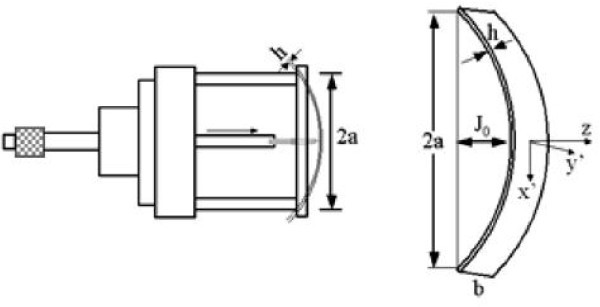
**Schematic drawing of the uniaxial strain apparatus**.

In order to study the effect of uniaxial strain on the IPOA, we cleaved the sample with well width 5 nm into a 25 × 4 mm^2 ^strip. Uniaxial strain was introduced by a stress device as shown in Figure 1 which is the same as the one used by Papadimitriou and Richter [15]. When the length-to-width ratio is greater than 3, the strip behaves like a bend rod, and the apparatus produces only two nonzero strain components:*ϵ*_*x*'*x*' _(tensile) *ϵ*_z'z' _and (compressive). Here *x*' and *y*' are along the cleavage axis [110] and [110] as shown in Figure 1. Transformed to the principal axis [100] and [010], the nonzero strain components are *ϵ_xx_*, *ϵ_yy_*, *ϵ_zz _*and *ϵ_xy _*[4], and only *ϵ_xy _*will introduce IPOA. The maximum strain component *ϵ_xy _*at the center of the strip is given by [16]

Here *J*_0 _is the deformation at the strip center, *h *is the thickness and 2*a *is the length of the strip. The relative reflectance difference between the [110] and [110] directions at the center of the strip (3 × 4 mm^2^) is measured by RDS at room temperature.

## Results and discussion

### Experimental results

**Figure 2:**
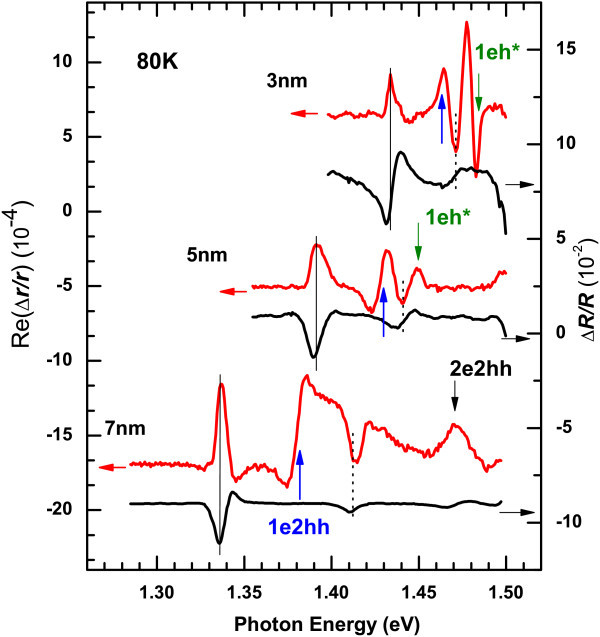
**Real part of RD spectra and *ΔR*/*R *spectra of In_0.2_Ga_0.8_As/GaAs single-quantum well with nominal well width 3, 5, and 7 nm, respectively**. The spectra are measured at 80 K. The vertical lines indicate the energy positions of the transitions 1e1hh (solid) and 1e1lh (dotted). And the vertical arrows indicate the positions of 1e2hh (upward arrows), leh* (downward arrows), and 2e2hh (downward arrows). Here h* represents continuous hole states.

Figure 2 shows the real part of the RD and *ΔR/R *spectra of the three samples obtained at 80 K. In the *ΔR/R *spectra, we can observe the transitions of 1e1hh (the first conduction to the first valence subband of heavy hole), 1e1lh and 2e2hh, and what's more, the intensity of the transition 1e1hh is much larger than that of the 1e1lh. However, in the RD spectra, besides the allowed transitions 1e1hh, 1e1lh, 2e2hh and 1eh*, we can also observe the forbidden transition 1e2hh. Here h* represents continuous hole states. The energy positions of the transitions 1e1hh (1e1lh) are marked by solid (dotted) lines. And the positions of 1e2hh, 1eh* and 2e2hh are indicated by upward, green downward and black downward arrows, respectively. The transitions 1e1hh and 1e1lh show peak-like lineshape (negative or positive), while the forbidden transitions 1e2hh of the samples with well width 5 and 7 nm present a smoothed-step-like lineshape. This phenomenon may be attributed to the coupling of heavy and light holes when the in-plane wave vector is nonzero [1]. For the sample with well width 3 nm, it is difficult to clearly distinguish the corresponding energy positions of the transitions 1e2hh, 1e1lh and leh*, because they are too close to each other. Even so, we can still observe that, the intensity of the IPOA of 1e1lh increases obviously compared to that of 1e1hh. Surprisingly, the forbidden transition 1e2hh are comparable to the allowed transition in RD spectra, while it almost cannot be observed in *ΔR/R *spectra.

**Figure 3:**
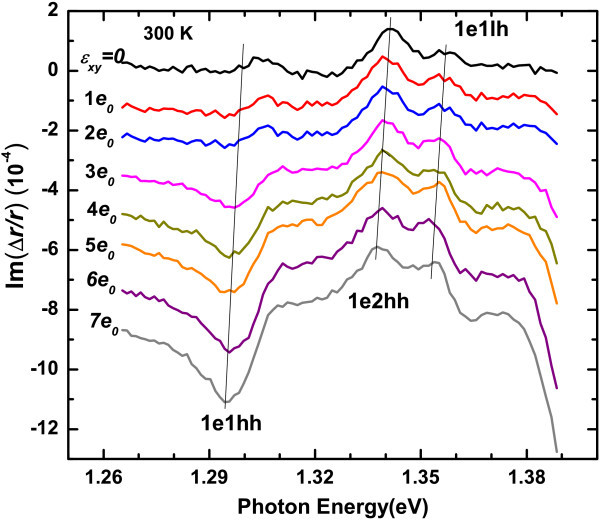
**RD spectra of 5 nm-In_0.2_Ga_0.8_As/GaAs QW under different strain *ϵ_xy _*in unit of *e*_0 _= 3.23 × 10^-5^**. The spectra are measured at room temperature and shifted vertically for clarity. The oblique lines indicate the energy positions of the transitions 1e1hh, 1e2hh, and 1e1lh in the RD spectra.

**Figure 4:**
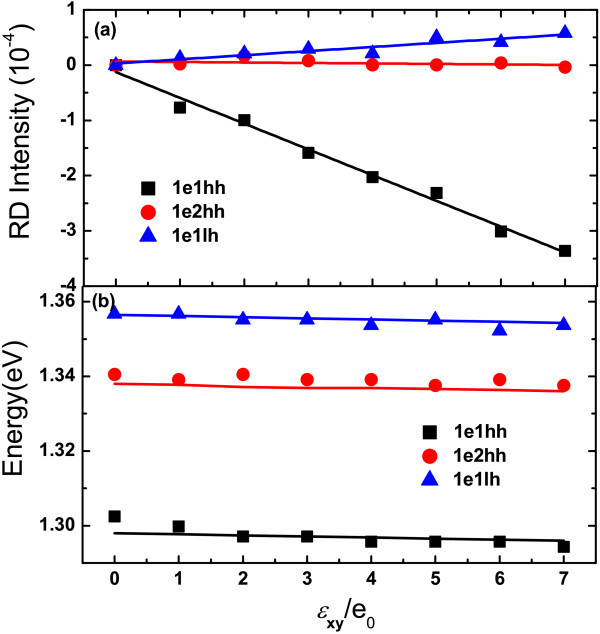
**Strain dependence of RD intensity and energies of 1e1hh, 1e2hh and 1e1lh**. **(a)** RD intensity of the transitions 1e1hh (squares), 1e2hh (circles) and 1e1lh (triangles) vs. strain after subtracting the RD contribution under zero strain. The solid lines are the linear fitting of the experimental data. **(b) **The transition energies vs. strain. The solid lines in **(b) **are calculated from the envelope function theory (1*e*_0 _= 3.23 × 10^-5^)

Figure 3 shows the imaginary part of RD spectra of the sample with 5 nm well width under different strain. Although the signal-to-noise ratio at room temperate is not as good as that at 80 K, three structures can still be clearly observed in the vicinity of 1.30, 1.34 and 1.36 eV, which can be assigned to the transitions of 1e1hh, 1e2hh and 1e1lh, respectively. Figure 4a shows us the RD intensity of the transition 1e1hh, 1e2hh and 1e1lh vs. strain, after subtracting the RD contribution under zero strain. It can be seen that, as the strain increases, the RD intensity of the allowed transition 1e1hh and 1e1lh are enhanced, while that of the forbidden transition 1e2hh does not show apparent change. Besides, in contrast to the transition 1e2hh and 1e1lh, the sign of the anisotropic transition 1e1hh changes as the strain increases. In addiction, slight redshifts can be introduced by the strain for all transitions, as shown in Figure 4b. The energy shift caused by *J*_0 _= 0.07 (i.e., *ϵ_xy _*= 7*e*_0 _= 2.3 × 10^-4^) is less than 9 meV.

### Models and calculation results

It is well known that, IPOA in (001)-oriented QWs mainly comes from mixing between heavy and light holes [2,3,17]. However, it is demonstrated that the spin-orbit coupling has significant effects on the band structure especially for highly strained quantum wells [18]. The strain will couple the heavy-hole (hh) bands, light-hole (lh) bands with spin-orbit split-off (SO) band [18]. Therefore, taking into account the coupling between hh, lh and SO band, we use 6 band **K · P **theory which is described in Ref. [18], and treat the hole-mixing induced by the strain *ϵ_xy_*, electric field and the two interface as perturbation [4]. The perturbation Hamiltonian *H*' can be written as [18](1)

with [2,4](2)

and [18](3)

for the basis |3/2, 3*/*2* >*, |3*/*2, 1/2* >*, |3/2, -1/2* >*, |3*/*2, -3/2* >*, |1/2, 1/2* >*, and |1*/*2, -1/2* >*. Here *b *and *D *are the Bir-Pikus deformation potentials, *F *is the electric field along the z direction, *d*_14 _is the piezoelectric constant, *ϵ_ij _*denotes the symmetric strain tensor, *P*_1 _(*P*_2_) is the lower (upper) interface potential parameter describing the effect of *C*_2ν _interface symmetry [2], *l*_1 _(*l*_2_) is the In segregation length in the lower (upper) interface, and *z *= ±*w*/2 is the location of the interfaces of QW. The interface potential parameter *P*_1 _and *P*_2 _are equal for a symmetric QW, and anisotropic interface roughness will make them unequal [4]. According to the model suggested in Ref. [19], we assume that the segregation lengths on the two interfaces are equal, i.e., *l*_1 _= *l*_2_.

In order to estimate the value of built-in electric field, we perform photoreflectance measurements. However, no Franz-Keldysh oscillations presents, which can be attributed to the fact that the layers are all intentionally undoped and the residual doping is very low. Thus, the residual electric field is weak enough to be neglected.

Based on the Luttinger 6 × 6 hole Hamiltonian [18] and the hole-mixing Hamiltonian described in Equation 1, the energies of *n*e-*m*lh/hh transition and transition probability can be calculated. Then using a Lorentzian function, as described in Equation 4, we can simulate anisotropic transition spectroscopy *ΔM *and average transition spectroscopy *M*.(4)

here *Γ *is the linewidth of the transition, and *E_nm _*(*P_nm_*) is the transition energy (probability) between *n*e and *m*lh or between *n*e and *m*hh. In the calculation, the adopted Luttinger parameters are: *γ*_1 _= 6.85, *γ*_2 _= 1.9, *γ*_3 _= 2.93 for GaAs, and *γ*_1 _= 21.0, *γ*_2 _= 8.3, *γ*_3 _= 9.2 for InAs. The band-offset is taken as *Qc *= 0.64 [20], and the strain-free In*_x_*Ga_1-*x*_As band gap at 80 and 300 K are taken from Refs. [20] and [21], respectively. The other band parameters are got from Ref. [22]. The anisotropic transition probability *ΔM *is proportional to *Δr/r*. Therefore, we can compare the theoretical calculated *ΔM *with experimental data *Δr/r*, and thus to find out the reason responsible for the observed strong anisotropic forbidden transitions. It is noteworthy that even under zero uniaxial strain, there will still be residual anisotropic strain exists, which may be due to a preferred distribution of In atoms [23]. In the following, we will discuss the interface potential, segregation and anisotropic strain effect separately.

We should first estimate the value of interface potential parameter, denoted as *P*_0_. So far, there are four theoretical models estimating the value of *P*_0_: boundary conditions (BC) model by Ivchenko [17], perturbed interface potential model (called "*H_BF_*") by Krebs [3], averaged hybrid energy (AHE) difference of interfaces model and lattice mismatch model by Chen [24]. Given that BC model is equivalent to *H_BF _*model, we need to consider only one of them [24]. Thus using *H_BF _*, AHE and lattice mismatch model and then adding them up, we obtain the value of *P*_0 _is about 600 meV Å.

**Figure 5:**
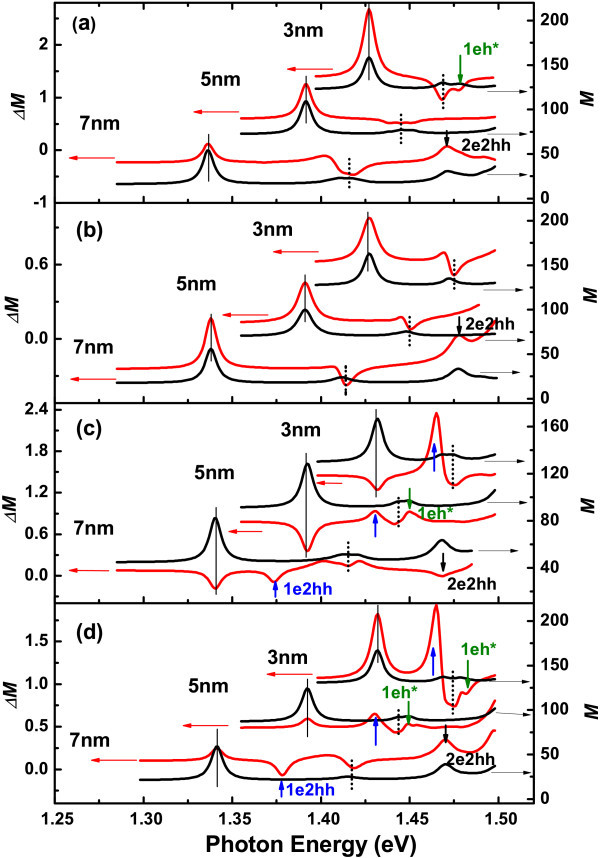
**Calculated anisotropic transition probability *ΔM *and average transition probability *M *of In*_x_*Ga_1-*x*_As/GaAs QW with well width 3, 5 and 7 nm, respectively**. The optical anisotropy is induced by **(a) **anisotropic interface structures, **(b) **anisotropic strain effect, **(c) **In segregation effect and **(d) **both anisotropic interface structures and In segregation effect. The vertical lines indicate the energy positions of the transitions 1e1hh (solid) and 1e1lh (dotted). And the vertical arrows indicate the positions of transitions 1e2hh (upward arrows), leh* (downward arrows), and 2e2hh (downward arrows).

If there is only anisotropic interface structures in the interface, i.e., *l *= 0, *ϵ_xy _*= 0, we can adopt *P*_1 _= *P*_0_, and fit *P*_2 _to the experimental data. The fitting results are shown in Figure 5a. The *P*_2 _value adopted is 775 meV Å. It can be seen that, only the allowed transition presents. Therefore, the observed anisotropic forbidden transition cannot be attributed to anisotropic interface structures.

If there is only anisotropic strain effect in the QW (i.e., *P*_1 _= *P*_2 _= *P*_0_, *l *= 0), only one free parameter *ϵ_xy _*can be fitted to the experimental data. The fitting result is shown in Figure 5b. The *ϵ_xy _*value we adopt is 0.003 × *ϵ_xx _*= *-*4.24 × 10^-5^. Again, there is no forbidden transition presents. Therefore, the observed anisotropic forbidden transition cannot be attributed to anisotropic strain effect.

If there is only atomic segregation effect (i.e., *P*_1 _= *P*_2 _= *P*_0_, *ϵ_xy _*= 0), one can fit free parameter *l *to the experimental data. The fitting result is shown in Figure 5c. The fitted segregation length *l *is 1.8 nm, which is in reasonable agreement with that reported in Ref. [19]. Apparently, the segregation effect will lead to a strong IPOA for the forbidden transition 1e2hh, but do not change its average transition probability, which is still very small. Besides, for the sample with well width 3 nm, a strong IPOA is also present for the transition 1e1lh. Therefore, the observed anisotropic forbidden transition is closely related to In atomic segregation effect.

From Figure 5c, we can see that, if there is only segregation effect, the sign of the transition 1e1hh is negative, which is not consistent with the experiment. Therefore, there must be some other effect existing, such as anisotropic interface structures or anisotropic strain effect. When we take both the anisotropic strain and segregation effect into account, the calculated results are not consistent with the experimental data. However, the results obtained by both the anisotropic interface structure and the segregation effect are in reasonable agreement with the experiment, as shown in Figure 5d. In the calculation, we adopt interface parameter *P*_1 _= 595 meV Å, *P*_2 _= 775 meV Å, and the segregation length *l *= 1.8 nm. The obtained interface potential difference *ΔP/P*_0 _is about 30%, which is much larger than that obtained in GaAs/Al*_x_*Ga_1-*x*_As QW (about 6%) [4]. The reason may be that lattice mismatch will enhance the interface asymmetry of the QWs.

**Figure 6:**
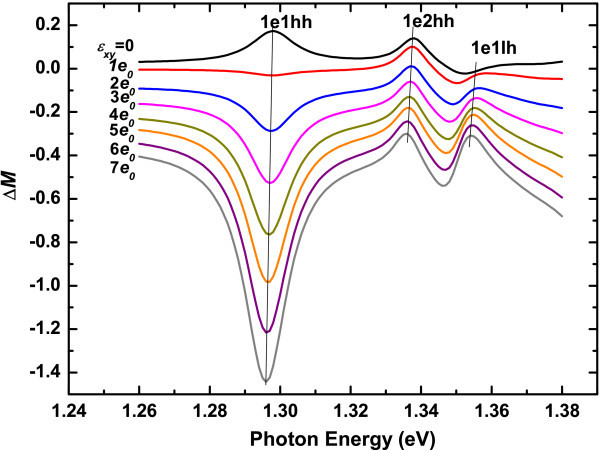
**Calculated anisotropic transition probability *ΔM *of In*_x_*Ga_1-*x*_As/GaAs QW under different strain *ϵ_xy _*in unit of *e*_0 _= 3.23 × 10^-5^**. The oblique lines indicate the energy positions of the transitions 1e1hh, 1e2hh, and 1e1lh in the *ΔM *spectra.

Using the parameters obtained above, we can well stimulate the IPOA of all the transitions under different uniaxial strain, as shown in Figure 6. The calculated transition energies are also well consistent with experiments, which is shown in Figure 4b.

### Interpretation of IPOA by perturbation theory

The IPOA-intensity ratio of 1e1lh and 1e1hh transitions is much stronger for the sample with 3 nm well width compared to that of the other samples. This phenomenon may be undefirstood in the following way. According to perturbation theory, the anisotropic transition probability *ΔM *of 1e1lh can be expressed as [1,2](5)

Here 〈1*E*|*nH*〉 is the overlap integral between the first electron and the *n*th heavy-hole states. 〈1*H*|*R*(*z*)|1*L*〉 is the hole-mixing strength between 1*H *and 1*L*. *E*_1*L *_- *E_nH _*is the energy separation between 1*L *and *nH*. It can bee seen that, *ΔM *is directly proportional to the coupling strength of holes and inversely proportional to their energy separation. For the three samples, there is little difference in the term *R*(*z*). However, *E*_1*L *_*- E*_2*H *_of the sample with 3 nm well width is smaller than that of the other samples, which results in much stronger IPOA.

The appearance of the forbidden transition and its behavior under uniaxial strain can be interpreted in a similar way. According to perturbation theory, the anisotropic transition probability *ΔM *of 1e2hh can be expressed as [1,2](6)

Here 〈1*E*|*nH*〉 and 〈*nH*|1*E*〉 (〈SO|1*E*〉) are the overlap integrals between the discussed electron and hole (SO) states. 〈1*L*|*R*(*z*)|2*H*〉 is the hole-mixing strength between 1*L *and 2*H*, and 〈2*H|R*(*z*)|SO〉 is the coupling strength between 2*H *and SO band. *E*_2*H *_- *E*_SO _is the energy separation between 2*H *and SO. Since *E*_2*H *_- *E*_SO _≫ *E*_2*H *_*- E*_1*L*_, the coupling between 1*L *and 2*H *dominates. When there is no segregation effect, 〈2*H*|1*E*〉 = 0 and no optical anisotropy exists. However, when segregation emerges, the symmetric square well changes into an asymmetric well, which will change the parities of the subband wave functions. Besides, it will also couples the 1*L *and the 2*H *subbands, and as a result, the perturbed 2*H *subband wave function now contains a small portion of the unperturbed |1*L*〉 one. Thus, 〈2*H*|1*E*〉 ≠ 0, and its value is proportional to the segregation effect. The strain component *ϵ_xy_*, being an even function of space, only couples the sub-bands with same parity, such as 1*H *and 1*L*. Then, the contribution of *ϵ_xy _*to the numerator of the first term in Equation 6 can be written as(7)

in which the first integral is nearly a constant, and 〈1*L*|2*H*〉 〈2*H*|1*E*〉 is mainly determined by the segregation effect and interface potential. Therefore, for the forbidden transition 1e2hh, the change of IPOA induced by a weak uniaxial strain (in the order of 10^-5^) will be too weak to be observed in experiment. However, for the allowed transitions, such as 1e1hh, the strain will also couple 1*H *and 1*L*, and will remarkably change the IPOA. From Figure 3 we can see that the RD intensity of transition 1e1lh does not show significant change as the strain increases. The reason may be that the light-hole band configuration is weak type I for the current alloy composition [20], which result in the change of the potential has little influence on its wave function.

## Conclusion

We have observed strong anisotropic forbidden transition in a series of In_0_._2_Ga_0_._8_As/GaAs SQW with well width ranging between 3 nm and 7 nm at 80 K. Using a 6 band **K **· **P **theory, we have calculated the optical anisotropy induced by interface composition profile due to In segregation, anisotropic interface structures and anisotropic strain. It is found that the observed anisotropic forbidden transition can be mainly attributed to the In segregation effect. Besides, the effect of uniaxial strain on IPOA is also investigated. It is found that the IPOA of the forbidden transition changes little with strain, while that of the allowed transition shows a linear dependence on strain. Finally, an interpretation of IPOA by perturbation theory is also given out.

## Abbreviations

AHE: averaged hybrid energy; BC: boundary conditions; In: indium; IPOA: in-plane optical anisotropy; SQW: single-quantum well; SRE: symmetry reduction effect; PR: photoreflectance; QW: quantum well; RDS: reflectance-difference spectroscopy.

## Competing interests

The authors declare that they have no competing interests.

## Authors' contributions

JLY performed the statistical analysis, carried out the calculations and drafted the manuscript. YHC conceived of the study, and participated in its design and coordination. CGT carried out the experiments. CYJ participated in the revision of the manuscript and discussed analysis. XLY participated in the design of the study. All authors read and approved the final manuscript.
